# Instrument to Identify Food Neophobia in Brazilian Children by Their Caregivers

**DOI:** 10.3390/nu12071943

**Published:** 2020-06-30

**Authors:** Priscila Claudino de Almeida, Beatriz Philippi Rosane, Eduardo Yoshio Nakano, Ivana Aragão Lira Vasconcelos, Renata Puppin Zandonadi, Raquel Braz Assunção Botelho

**Affiliations:** 1 Graduate Program in Human Nutrition, University of Brasília, 70910-900 Brasília, Brazil; beatrizphilippir@gmail.com; 2 Department of Statistics, University of Brasília, 70910-900 Brasília, Brazil; eynakano@gmail.com; 3 Department of Nutrition, University of Brasília, 70910-900 Brasília, Brazil; ivanaunb@gmail.com (I.A.L.V.); renatapz@yahoo.com.br (R.P.Z.)

**Keywords:** food neophobia, child, caregiver perception, instrument

## Abstract

This study aimed to develop a specific instrument to evaluate food neophobia focused on Brazilian children and to perform the content validation and internal semantic consistency and reproducibility evaluation of the instrument. Three steps were necessary to conduct the study: (i) development of the instrument, (ii) internal validation (content validation and semantic evaluation) of the food neophobia instrument using 22 experts in the first round and 20 of them in the second round, (iii) evaluation of the internal consistency and reproducibility of the instrument with the children’s caregivers, using the test–retest (where the same caregiver—*n* = 22—answered twice, with 24 h interval) and comparing responses between two caregivers (*n* = 44) of the same children (*n* = 22). We developed an instrument in Brazilian–Portuguese to evaluate children’s food neophobia based on the caregivers’ perceptions with 25 items divided into three domains (neophobia in general, neophobia for fruits and neophobia for vegetables). Our results indicated that the instrument has excellent internal consistency (>0.9) and reproducibility (>0.9) when answered by the caregiver who knows the child’s eating habits, indicating reliability to be applied in Brazil. In addition, when the two caregivers answered the instrument, we found a good reproducibility (>0.6), confirming the possibility to be answered by one of the caregivers. Further studies are necessary to complete external validation with a representative sample of the target group in Brazil, showing nationwide the profile of the population. The potential of a neophobia study would contribute to the implementation of effective strategies and guidelines to support parents and health professionals, especially those involved in health and nutrition, to identify traces of food neophobia or neophobic behavior. By accurately measuring food neophobia in children, families can prevent nutritional deficiencies throughout adolescence and adulthood, improving eating habits. Children usually have neophobias similar to the ones presented by their parents—and when early detected, these neophobias can be addressed.

## 1. Introduction

Globally, it is estimated that 131 million children aged between five and nine years, and 40 million under five years are overweight [1]. In Brazil, the rate of childhood obesity is eight times higher than childhood malnutrition [2]. Increasing obesity has been associated with the growing consumption of high energy density (ED) foods and poor nutritional quality diets [3].

Children displaying neophobic behavior tend to become overweight because their diet is often less varied and often deficient in fruit and vegetables. Additionally, the increase in neophobia may lead to the consumption of more energy dense foods with poor nutritional composition. Therefore, it is essential to find means to introduce healthy and nutritious foods to fight overweight and obesity to promote child’s health without triggering a neophobic response [4,5].

Food neophobia is the reluctance to eat or the avoidance of eating unfamiliar or novel foods, mainly healthier ones [6]. Most feeding difficulties are non-organic and without any underlying medical condition. Food neophobia is the resistance to the introduction of new food in a healthy child. It should be distinguished from real sensory food aversion or selective picky eating [7]. Food fussiness is the tendency to be highly selective about foods one is willing to eat and emerges in early childhood. Food neophobia is a closely related characteristic, being that these behaviors are associated [8].

Food neophobia is a common condition between children from two to three years old when they start a more adult-like diet and go through rapid changes and improvements in categorizing food [9]. Neophobia peaks between two and six years of age then decreases until it stabilizes in adulthood [6,10]. Food neophobia can be learned through parents’ food preferences [11].

This eating behavior prevalence and responses are variable around the world given the different age groups, instruments, cutoff points and respondents used in the studies [12,13,14,15,16]. However, it is estimated that the neophobia prevalence in individuals under 18 years old ranges from 40% to 60% [17]. A study with Polish preschool children (*n* = 325) showed that 10.8% of the children presented a high level of food neophobia and 76.9% medium level of neophobia [8]. Another study conducted with 200 mothers of under-five-year-old children from India found a neophobia prevalence of 37% among the children [16]. In Spain, a study used the food neophobia scale with 1057 primary schoolchildren (8–10 years old), showed 13.5% of food neophobia prevalence among the participants [17].

Despite the wide range of neophobia rates reported in studies, the high prevalence reported in most studies is worrying. It is not possible to compare the evaluated samples due to the different types of instruments used that are sometimes not even validated. In Brazil, there are still no studies with children that have analyzed neophobia prevalence, mainly because there is no developed instrument for this purpose that takes into account the country’s social and cultural reality. Tools adapted for each country are essential because nutrition recommendations can vary. In addition, the language and expressions differ among nations. The information must be well-described to guide the respondent to minimize bias.

For the development of an instrument, the phenomena of interest must be translated into concepts that can be measured, observed or recorded. Without proper methods for data collection, the validity of a given instrument´s conclusions is questionable. It is essential to consider the relevant literature; the clarity, consistency and relevance of each item; the evaluation of the instrument by relevant experts and the testing of the instrument to obtain the desired information [18,19]. An expert panel consensus helps to define the instrument items which should be maintained, revised or excluded and its application is increasing in several areas [20]. Another important procedure to obtain a satisfactory instrument is to perform the semantic evaluation, which measures the comprehension of the instrument items by the experts and helps to evaluate the need to rewrite the questions to achieve a better comprehension of the instrument [21]. To evaluate the instrument, before the application in a large sample, it is essential to test the reproducibility (reliability) and internal consistency with a pilot study [22,23,24,25].

Due to the lack of instruments on food neophobia for children in Brazil, leading to the lack of information about this Brazilian target group, this study aimed to develop a specific instrument to evaluate food neophobia among Brazilian children and to perform the content validation and semantic evaluation. In addition, internal consistency and reproducibility evaluation of the instrument was performed in a pilot study. We expect that this study can provide an instrument for assessing children’s food neophobia, making it possible to determine which types of food children are more reluctant to try.

## 2. Materials and Methods

Three steps were necessary for the study: (i) development of the food neophobia instrument, (ii) internal validation (content validation and semantic evaluation), (iii) evaluation of the internal consistency and reproducibility of the instrument with the children’s caregivers. The study was approved by the Health Sciences Ethics Committee, University of Brasilia, No. 3.339.807 and followed the guidelines established by the Declaration of Helsinki.

### 2.1. Development of the Questionnaire

The first part of the instrument presented items on the characterization of the sample (gender and age of the child, family income and respondent’s relationship to the child). The second part was specific to evaluate neophobia. Its construction was based on extensive literature review without the restriction of time and language for the choice of instruments. Questions were subject to adaptations, considering the use for Brazilian children. Therefore, the following instruments found in the literature review and validated were used to design the preliminary version of this research instrument: food neophobia scale (FNS) for adults [26], food neophobia test tool (FNTT) for children [12] and fruit and vegetable neophobia instrument (FVNI) aimed at children [27].

The first instrument, the food neophobia scale [26], was developed in Toronto, Canada, composed of 10 items scored on a seven-point scale. Male and female adult undergraduate students in psychology (from 18 to 74 years old) were evaluated. The second instrument, the food neophobia test tool [12], from Denmark, was composed of 19 items using a five-point Likert scale. The study applied the instrument with children from 9 to 13 years old, and it was based on a review of thirteen designs to assess food neophobia and willingness to try unfamiliar foods. The fruit and vegetable neophobia instrument, with 18 items [27], was developed in the United States and students, from 8 to 10 years old, answered it. The FVNI used a 4-point agreement scale [27].

We conducted the translation of these three instruments. After that, each item was carefully read and similar items were matched. The items that were not applicable to Brazilian children were removed. Specifically, the items that did not represent the Brazilian context or were not applicable for assessing eating behavior for children were removed. Items that indicated ethnic foods or restaurants, for example, were not included because there were no synonymous expressions and Brazil is a country with great food diversity and rich in cultural influences [28,29,30]. In addition, the FNTT [12] was developed to be answered by the child; therefore, the format of the items was changed to be answered by caregivers about their child. All of the 18 items of FVNI [27] (about fruits and vegetables) were used in our instrument, with adaptations.

In addition to the items from the previously mentioned instruments, four items were added by researchers because they considered them essential to evaluate the neophobia in children, and they were not identified in other instruments. The final instrument involved the following variables (I) food neophobia in different environments—home, friends’ house, school, social events- and situations—birthday parties, friends meetings and (II) food neophobia with an emphasis on fruits and vegetables. We chose to use a five-point scale as options to answer each item because studies have shown that it is an accurate scale to measure what it wants to measure with several possibilities that are not tiring for the participant [31,32,33,34]. The adaptation of items to the Brazilian context was necessary for the local reality. After the preliminary version of the instrument, we invited a panel of experts, impartial and with different levels of education (M.Sc., Ph.D. and postdoc) and expertise to judge the items regarding importance and comprehension.

### 2.2. Internal Validation of the Food Neophobia Instrument

#### 2.2.1. Subjective Evaluation

This study adopted the Delphi method to perform the semantic and content validation of the instrument. It is a widely used method for building a consensus, being a handy tool for diagnostic situations. The Delphi method is used in several types of studies, and it has gained popularity for the internal validation of instruments [35]. Generally, the purpose is to obtain a consensus among specialists on different issues. These specialists are individuals who understand the addressed subject and can contribute to the creation and validation of data collection instruments [36].

It is characterized by involving experts to assist with a wide range of opinions on a specific topic. The experts give their impression anonymously, allowing everyone to express their thoughts. Researchers give feedback on the same platform to communicate with the experts, minimizing possible biases [37]. Construct validity constitutes a direct way to verify the legitimacy hypothesis of the behavioral representation of latent traces [38].

Twenty-five experts were contacted by email and invited to participate; 22 of them agreed to participate in the first stage. As inclusion criteria, experts should have at least a master’s degree and experience in childhood nutrition, eating behavior or clinical nutrition. The experts’ mean age was 41-year-old, and three of them presented postdoc, five experts with Ph.D. and fourteen with a master’s degree. In the second stage, 20 experts participated, because two were unable to attend in this phase. The instrument was assessed using the Google Forms^®^ online platform, in which the experts electronically signed the consent form and proceeded to analyze the items. The experts were asked to express their opinion and to evaluate the preliminary version of the instrument, considering aspects such as the content, clarity, type and consistency of the items. Experts were also asked to suggest any modification, exclusion or inclusion of items they judged as relevant. Additionally, they could freely comment on any subject regarding the instrument.

For the initial round, the instrument was available with all questions on an online platform to guarantee anonymity. The online survey contained the necessary information on the topic, instructions for filling up and specific spaces for opinions on changes or exclusion of items—as well as other information that experts judged importantly. They could suggest items or replacements, as well as change the order of the questions, using the final suggestions field [37]. Experts were asked to evaluate each item considering its importance for the instrument, using a Likert scale from (1) “I fully disagree with the item” to (5) “I fully agree with the item”. We also used the Google forms^@^ platform to provide feedback to the experts in regard to the evaluations performed by other experts and the final results of the analysis. Items not approved in a stage were presented to the experts so that opinions could be shared. After being informed about the other experts’ opinions, the experts were asked to review their analysis and decide whether or not they would confirm their previous answers. This procedure was performed to obtain a consensus among the experts.

The criteria established for the approval of the item was a minimum of 80% agreement among the experts (W-values ≥ 0.8) [35]. Additionally, items should have had a mean ≥ 4 for the evaluation of importance (content validation) and clarity (semantic evaluation) to be maintained in the instrument. Items not considered essential for the instrument were excluded. Unclear items were rewritten in a different manner and subject to further evaluation by the experts. Suggestions made by the experts were considered and incorporated into the final version of the instrument.

If an item was not approved, the criterion for the exclusion was the expert feedback. Each observation was cautiously read to understand if the item could be improved and restructured.

Therefore, the exclusion of questions was the last option, following as many steps as necessary for the item to be approved by the experts. If the item distanced itself from the original meaning of the question because there were no pertinent suggestions or it lost its purpose or did not represent a neophobic behavior, then exclusion was an option. If the item had a high percentage of recommendations for elimination, it was excluded.

#### 2.2.2. Content Validation and Importance

Experts evaluated each item considering its importance for the food neophobia in Brazilian children. A Likert scale was used, wherein: (1) “I totally disagree with the item”; (2) “I partially disagree with the item”; (3) “I neither agree or disagree with the item”; (4) “I partially agree with the item”; and (5) “I totally agree with the item”. Items with 80% or more of approval did not require further evaluation or reformulation [35]. An assessment of importance was also conducted with a question about whether or not the item should be excluded. The options for this response were yes or no. Questions not approved by the experts took into account the suggestions to be reformulated or eliminated according to their evaluation.

#### 2.2.3. Semantic Validation

The semantic evaluation of the instrument was performed simultaneously with the content validation, using the same survey in the Google Forms^®^. Experts evaluated each item regarding its clarity and considering their level of understanding of the subject. The Likert scale was used with the following options: (1) “I did not understand it at all”; (2) “I understood it a little”; (3) “I understood more or less”; (4) “I understood almost everything”; (5) “I understood it perfectly and had no questions”. Items considered unclear, without 80% of approval, were reformulated, differently rewritten, considering the experts’ suggestions. After that, they were revaluated by the experts [35].

### 2.3. Evaluation of the Internal Consistency and Reproducibility

For the analysis of internal consistency and reproducibility, parents of twenty-two children aged four to eleven years answered the final instrument. This was a convenience sample, with the invitation of parents from different states of Brazil. After parental acceptance, they received the instrument through the Google Forms^®^, an online platform, with all the instructions for completing it, as well as the informed consent form. On the same day, both parents received the invitation, and they were asked to answer the instrument independently without receiving help from family members. Parents answered the approved instrument anonymously, with the child being identified by age and name initials. Each parent was instructed to answer according to his/her perception, without any help and without asking the child for best answer. For the analysis between individuals, both respondents received the instrument on the same day.

Food preferences continue changing throughout life and for young children, it is a rapid dietary change [39]. Therefore, 24 h after the first filling up by one of the caregivers, he or she was asked to answer the same instrument again. With two responses from the same caregiver, the intraindividual analysis was performed. This step was necessary to evaluate the reproducibility of the questionnaire. It served to verify possible difficulties about the context and understanding of the instrument once the guardian answers the instrument and not the child.

Reproducibility was tested considering the items of the instrument that, in one of the extremes of the scale, pointed to a probably neophobic behavior. These joint items create a score. Lower values scored in the instrument present higher chances of the child having a neophobic behavior.

In addition to the score, domains were created to allow a more sophisticated assessment of neophobic behavior. Three dietitians were responsible for discussing each item of the instrument to create the domains and classify the questions in just a single domain. This was taken into consideration which subject stood out because, in the same question, there could be different essential aspects of food neophobia, for example, different places (house, friends’ houses, school) and fruits. Dietitians sought for a balance among the items in each domain.

#### Participants

The children whose (*n* = 22) caregivers participated in the test–retest stage were mostly male (59%, *n* = 13) than females (41%, *n* = 9), mean age of 6.72 ± 2.35 years old. Among the caregivers, 22 were mothers (mean age 36.89 ± 9.12 years), and 22 were fathers (mean age 38.60 ± 9.95 years). All of the participants lived with their children.

### 2.4. Statistical Analysis

For the analysis, the data were extracted from the Google Forms^®^ platform in a Google^®^ spreadsheet and analyzed using the SPSS^®^ 25.0 software, using descriptive statistics and presented as mean and standard deviation, frequencies and percentages.

For the content and semantic validation of the instrument, the approval percentage of each item was calculated to assess the importance, clarity and its degree of understanding. The evaluated items should have at least an 80% agreement [35].

The reliability of the instrument in general and the adequacy of each domain was determined by internal consistency. Internal consistency of the entire instrument was performed by analyzing Cronbach’s alpha [38], as well as its domains. The minimum acceptable value for a reliable questionnaire is 0.7; from ≤0.8 to <0.9, the result is considered good, and α ≥ 0.9 is considered an excellent value [40]. In this step, the instrument was answered three times for each child. One caregiver answered two times (without previous knowledge about the need to answer the second time), and the other caregiver answered once. Answers from the first of the three completed instruments for each child were considered to calculate Cronbach’s alpha.

Reproducibility was assessed intraindividual (the same caregiver answered the instrument at two different times for the same child) and between individuals (two caregivers answered the instrument for the same child) by the intraclass correlation coefficient (intraclass correlation coefficient —ICC). Values equal to or greater than 0.6 to 0.74 indicates a good level of reproducibility and above 0.75, excellent [41].

## 3. Results

### 3.1. Construction of the Instrument, Content Validation and Semantic Evaluation

**Figure 1:**
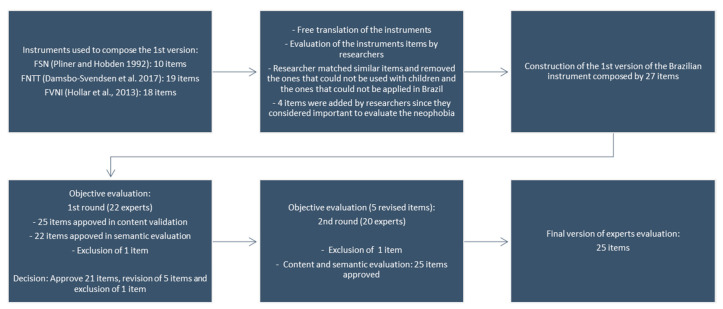
Summary of the construction, content validation and semantic evaluation process of the food neophobia instrument in children in Brazilian–Portuguese.

In the first stage of experts’ evaluation, from 27 items, 21 items (80.6%) were approved by content and semantic evaluation, and one was excluded. The suggestions for the nonapproved items were revised, and five items were rewritten to be reevaluated by experts. Two stages of modifications were necessary until the approval of the final version of the instrument (Figure 1). After all the changes indicated in the first stage, experts judged the five items not approved in the first step. Experts indicated one item to be excluded and approved 25 items to the final version of the instrument. Therefore, the final version of the Brazilian food neophobia in children instrument (Appendix A) was sent to the evaluation of reproducibility and internal consistency.

### 3.2. Internal Consistency and Reproducibility

After the evaluation by experts, the instrument was applied in a sample of children’s caregivers to evaluate internal consistency and reproducibility. At this stage, the responses of the instrument were compared between the two applications of the same child caregiver (test–retest) and responses between two caregivers of the same child.

Twenty-five items composed the score of the final instrument, divided into three domains (Table 1). The first domain was classified as general neophobia, to cover items that approach food neophobia in different environments that the child is not used to, such as a friend’s house or school. The second one was related to items that address fruits in the food context. The last domain was composed of items regarding the context of vegetables.

Nine items composed the first domain (1, 2, 3, 4, 5, 22, 23, 24 and 25). The second domain was composed of eight items (6, 7, 10, 11, 12, 13, 14 and 15) as well as the third one (8, 9, 16, 17, 18, 19, 20 and 21). The domains were well balanced, with a similar number of items, allowing better analysis when assessing the score of the whole instrument and also for each domain.

Reproducibility was verified considering the total score of the instrument and also the scores of each domain (Table 1). The scores were defined as the sum of the values of each item. Therefore, the score for the first domain may vary from 9 to 45 and the score for the second and third domains from 8 to 40. The overall score of the instrument may range between 25 and 125. Lower values indicate high neophobic behavior. All domains and the complete instrument presented excellent internal consistency (α > 0.9) and excellent intraindividual reproducibility (ICC > 0.9) (the same person answering the instrument twice). It indicated that the instrument is consistent and replicable. Reproducibility between individuals (two caregivers answering the instrument for the same child), was good (ICC > 0.6). All *p* values were statistically significant. As expected, intraindividual reproducibility was better than between two caregivers.

The items of the final instrument were divided into three domains. The first domain was classified as neophobia in general, to cover items that approach food neophobia for different environments that the child is not used to, such as a friend’s house or school. Schools can often be a strange environment at the beginning for younger children, and these often change depending on the school grade. The second domain was directed to items that address fruits in the food context. Additionally, the third and last domain was composed of items that addressed vegetables. According to statistical analysis, the creation of the score and domains was accurate. There was no need to change the items among the domains, neither to balance the quantity. Reproducibility was verified considering the total score of the instrument and also the scores of each domain (Table 1). The domains enable an assessment of food neophobia in general when the whole instrument is used. However, it can assess for fruits and vegetables, for example, when domains two and three are used.

## 4. Discussion

This study is the first to develop and perform the internal validation of a neophobia instrument to evaluate children in Brazil. Assessing food neophobia in children may contribute to indicate how varied or restricted the diet is, allowing interventions to minimize the effects of a monotonous diet, frequently low in nutrients content [4]. To our knowledge, there are versions of the food neophobia scale carried out in Canada [26], China [42], Denmark [12], United States [43], Spain [44], Italy [45], among others, with none performed in Latin America focused on children.

Our instrument was constructed based mainly on three instruments [12,26,27] with adaptations for the language (Brazilian–Portuguese) and culture (Appendix A).

Two of the three used instruments (FNTT and FVNI) [12,27] focused on children. Each item was evaluated, similar items matched and items not applicable to Brazilian children were removed. Several additional items considered to be essential to the evaluation of neophobia in children were added in this study.

There is no consensus about the number of experts necessary to evaluate the instrument. In Brazil, Pasquali [38] states that six is the minimum, varying according to the instrument. However, there is a consensus that the number of experts cannot be too small and too few to hinder the existence of a consensus [37].

After the expert analysis, twenty-five items composed the score of the final instrument. Each item had precisely the same importance. Therefore, no issue was highlighted or considered as the key element. Each question had the possibility of having punctuation between one and five points. Considering the valid items that compose the instrument, its general score can vary from 25 to 125. In the internal validation, there is no gold standard nor a large enough sample to state the cutoff point of the score, not allowing, at this moment, to classify the neophobia as low, medium or high. In this sense, further studies with a representative sample of the Brazilian population target-group are necessary to define the cutoff point to best use the instrument.

There is a wide variety of administration intervals used in test–retest and equivalence studies seen within the literature. A systematic review on test–retest reliability showed that one percent of the studies had an interval of one hour or less, 18% had an interval of one day to one week, 25% had an interval of one week to two weeks, 21% had an interval of two weeks to one month, nine% had an interval of one to two months, 13% had an interval of two months or over and 13% reported a varied interval [46]. Considerations around the appropriate administration interval should be based on, among other things, an assessment of the stability of the condition involved and the complexity of the study sample [46]. According to Anastasi and Urbina [47], test–retest correlations decrease progressively as the interval lengthens. Especially for children, time for a second response should consider the cumulative effects reflecting changes in scholastic aptitude, mechanical comprehension, artistic judgment in addition to individual’s own home, school, community environment and other reasons such as illness or emotional disturbance. Therefore, in checking test–retest reliability, an effort to keep this interval shorter in children than for older persons should be made. The reproducibility performed with a short period (24 h) from the first and second responses for the same individual is interesting because the child changes his/her eating behavior throughout life, especially in the early years of childhood [39]. Collecting the data over a more extended period could show not representative results.

Responses of both caregivers were compared to determine if both responses could be used. Results showed that independently of the caregiver, the response is similar, showing good reproducibility. However, because we asked them not to check answers with the other caregiver, lower values were expected for analysis between individuals, the perception can be different. In addition, the time and activities with their child can be different. Our interpretation is that the third filling up of the instrument used to carry out the intraindividual analysis was answered by the caregiver who best knows the child’s eating behavior. Most of this analysis obtained more responses from mothers than from fathers. In general, in Brazil, mothers assume a social role as organizer and manager of domestic activities (including children’s food), presenting a more accurate knowledge about their child’s eating habits [48].

Despite the good reproducibility between different caregivers, our data showed that there is a difference when different people answer to the instrument. Hence, researchers recommend that the caregiver who best knows the child’s eating behavior responds to the instrument and does not check the child about the best response so that the answer is as reliable as possible. This point is essential, highlighting that the choice of the respondent has a direct impact on the assessment and may underestimate or overestimate food neophobia. In this sense, for children who spend the day at school, it may be necessary to check the school caregiver of the child, for example [49].

Previato and Behrens [50] translated to Brazilian–Portuguese the original version of FNS, evaluating adults in Brazil (*n* = 40). The authors also performed reproducibility by the intraclass correlation coefficient (ICC), ranging from 0.266 and 0.815 (*p* < 0.05). Our study presented better results for the reproducibility, 0.987 (*p* < 0.001). The internal reliability of the original scale for adults evaluated by Cronbach’s alpha coefficient reached 0.916, which demonstrates high reliability, similar to our results (0.958; *p* < 0.001). This version of the scale served as a basis for the same authors in 2017 to investigate the association of taste-related factors and food neophobia with nutritional status and food choices among Brazilian teenagers [50].

The original FNS [26] applied in adults (18–74 years old) showed the alpha coefficient for the food neophobia scale of 0.88. Our instrument presented a slightly higher alpha. An excellent alpha is a great result, as our instrument selected questions from the first food neophobia scale and other instruments.

The instrument can contribute to identifying which situations the child tends to have traces of food neophobia and for which foods in general or for fruits and vegetables that tend to be the foods that children most dislike.

The domains of the instrument were well balanced, with a similar number of items in each one, allowing better analysis when assessing the score of the general instrument and also for each domain. It is important to highlight that this instrument allows the assessment of children’s eating behavior for fruits, vegetables and preparations in general, thus being complete and not confused with a neophobia for certain foods. The instrument can facilitate the identification of traces of food neophobia, allowing interventions in childhood, which, when properly conducted, tend to be more efficacious [51].

## 5. Conclusions

This study developed an instrument in Brazilian–Portuguese with 25 items divided into three domains (neophobia in general, neophobia for fruits and neophobia for vegetables), to evaluate children’s food neophobia based on the caregivers’ perceptions. Our results indicated that the instrument has excellent internal consistency and reproducibility when answered by the caregiver who knows the child’s eating habits, showing to be reliable for application in Brazil. In addition, a good reproducibility was found when the two caregivers answered the instrument, confirming the possibility to be answered by one of the caregivers. Further studies are necessary to external validation with a representative sample of the target group in Brazil, showing nationwide the profile of the population. With the use of this instrument, other studies can discover the percentage of food neophobia in Brazil among children. It will be possible to differentiate the type of food neophobia and score the level of neophobia among children of different ages. Potentially with these answers, new studies can contribute to the implementation of effective strategies to support parents and health professionals to identify traces of food neophobia or neophobic behavior. By identifying the specific ages that present more neophobia and the foods that are more neophobic, caregivers or health professionals can establish priorities to deal with this group.

## Figures and Tables

**Table 1:** Measures of internal consistency and intraindividual reproducibility and between individuals, according to each domain of the instrument. Brazil, 2020. (*n* = 22).

Internal consistency *		Domain 1 (9 Items)	Domain 2 (8 Items)	Domain 3 (8 Items)	General (25 Items)
	Cronbach’s α	0.908	0.915	0.948	0.958
	*p*	<0.001	<0.001	<0.001	<0.001
Intraindividual reproducibility (the same individual answered twice)	Measure 1 Mean (SD)	24.82 (8.25)	22.05 (7.25)	20.91 (7.59)	67.77 (20.53)
	Measure 2 Mean (SD)	24.86 (7.88)	21.82 (7.31)	20.41 (7.32)	68.09 (19.97)
	ICC	0.983	0.978	0.979	0.987
	*p*	<0.001	<0.001	<0.001	<0.001
Reproducibility Between individuals (two different individuals answered the same questionnaire)	Measure 1 Mean (SD)	24.82 (8.25)	22.05 (7.25)	20.91 (7.59)	67.77 (20.53)
	Measure 2 Mean (SD)	24.95 (6.92)	20.68 (6.09)	19.50 (6.02)	65.14 (17.32)
	ICC	0.606	0.719	0.726	0.712
	*p*	0.022	0.003	0.002	0.003

* Internal consistency was calculated considering the first response of the instrument independent of the caregiver.

## Appendix A. Final Version of the Instrument in Brazilian–Portuguese and Free Translation to English

Neofobia alimentar em crianças (NAC)/*Food Neophobia in Children*

Prezado responsável, agradecemos muito se puder participar de uma breve pesquisa sobre neofobia alimentar em crianças. Responda sem o auxílio da crianças ou de outro responsável da família. Responda conforma a escala disponível no questionário variando de discordo totalmente a concordo totalmente ou nunca a muito dependendo da pergunta. *Dear caregiver, we thank you to participate of this brief survey about food neophobia in children. Please, answer without the help of the child or other caregivers. Use the scale available inside the questionnaire varying from totally disagree to totally agree or never to a lot according to the question.*

Idade da criança/*age of the child*:
Sexo da criança/gender of the child:
Grau de parentesco do respondente com a criança/*degree of kinship level of caregiver*:
Idade do respondente/*age of the caregiver*:
Sexo do respondente/gender of the caregiver:
Renda familiar bruta em salários mínimos/*Family income in* minimum *wages*:

Nos itens com o termo “hortaliças” considere todos os vegetais, com exceção de batatas, mandioca, inhame, cará e yacon/In the items which the name “vegetable” appear, do not consider starchy-vegetables (e.g., potato, cassava, yam, yakon).

**Item/*Item***	**Escala/*Scale***				
1. Meu (minha) filho(a) está disposto(a) a provar alimentos que nunca comeu antes *My child is willing to taste foods that he/she never tasted before*	Discordo totalmente *Totally disagree*	Discordo *Disagree*	Indiferente *Indifferent*	Concordo *Agree*	Concordo totalmente *Totally agree*
2. Se meu (minha) filho (a) sabe o que tem na comida, ele/ela irá prová-la *If my child knows what is in the food, he/she will taste it*	Discordo totalmente *Totally disagree*	Discordo *Disagree*	Indiferente *Indifferent*	Concordo *Agree*	Concordo totalmente *Totally agree*
3.Em eventos (reuniões, festas, etc), ele/ela prova novos alimentos *At meetings, parties, etc., he/she tastes new foods*	Discordo totalmente *Totally disagree*	Discordo *Disagree*	Indiferente *Indifferent*	Concordo *Agree*	Concordo totalmente *Totally agree*
4. Ele/ela não tem medo de comer alimentos que nunca experimentou antes *My child is not afraid to eat food that he/she never tasted before*	Discordo totalmente *Totally disagree*	Discordo *Disagree*	Indiferente *Indifferent*	Concordo *Agree*	Concordo totalmente *Totally agree*
5. Ele/ela acha divertido provar alimentos que nunca experimentou antes *My child has fun tasting foods he/she has never tried before*	Discordo totalmente *Totally disagree*	Discordo *Disagree*	Indiferente *Indifferent*	Concordo *Agree*	Concordo totalmente *Totally agree*
6. O quanto você acredita que seu (sua) filho (a) gostaria de frutas que ele/ela nunca experimentou? *How much do you believe your child would like fruits* *that he/she never tried?*	Nada *Not at all*	Pouco *Slightly*	Indiferente *Moderately*	Razoavelmente *Very*	Muito *Extremely*
7. O quanto você acredita que ele/ela gosta de provar frutas novas *How much do you believe he/she likes to taste new fruits?*	Nada *Not at all*	Pouco *Slightly*	Indiferente *Moderately*	Razoavelmente *Very*	Muito *Extremely*
8. O quanto você acredita que seu (sua) filho (a) gostaria de hortaliças que ele/ela nunca experimentou? *How much do you believe your child would like vegetables that he/she has never tried?*	Nada *Not at all*	Pouco *Slightly*	Indiferente *Moderately*	Razoavelmente *Very*	Muito *Extremely*
9. O quanto você acredita que ele/ela gosta de provar hortaliças novas? *How much do you believe he/she likes to taste new vegetables?*	Nada *Not at all*	Pouco *Slightly*	Indiferente *Moderately*	Razoavelmente *Very*	Muito *Extremely*
10. Você acha que ele/ela provaria uma fruta se ele/ela não souber o que é? *Do you think he/she would taste a fruit if he/she does not know what it is?*	Com certeza não *Certainly not*	Provavelmente não *Probably not*	Talvez *Maybe*	Provavelmente *Probably*	Com certeza *Certainly*
11. Meu (minha) filho (a) aceitaria provar uma fruta com aparência diferente do que está acostumado (a) a ver: *My child would taste a fruit that looks different from what he/she is used to see:*	Com certeza não *Certainly not*	Provavelmente não *Probably not*	Talvez *Maybe*	Provavelmente *Probably*	Com certeza *Certainly*
12. Você acha que ele/ela provaria uma fruta que ele/ela nunca provou antes? *Do you think he/she would taste a fruit that he/she never* *tasted it before?*	Com certeza não *Certainly not*	Provavelmente não *Probably not*	Talvez *Maybe*	Provavelmente *Probably*	Com certeza *Certainly*
13. Na casa de um amigo, você acha que ele/ela provaria uma fruta nova? *At a friend’s house, do you think he/she would taste a* *new fruit?*	Com certeza não *Certainly not*	Provavelmente não *Probably not*	Talvez *Maybe*	Provavelmente *Probably*	Com certeza *Certainly*
14. Na escola, você acha que ele/ela provaria uma fruta nova? *At school, do you think he/she would taste a* *new fruit?*	Com certeza não *Certainly not*	Provavelmente não *Probably not*	Talvez *Maybe*	Provavelmente *Probably*	Com certeza *Certainly*
15. Em casa, você acha que ele/ela provaria uma fruta nova? *At home, do you think he/she would taste a* *new fruit?*	Com certeza não *Certainly not*	Provavelmente não *Probably not*	Talvez *Maybe*	Provavelmente *Probably*	Com certeza *Certainly*
16. Você acha que ele/ela provaria uma hortaliça se ele/ela não souber o que é? *Do you think he/she would taste a vegetable if he/she does not* *know what it is?*	Com certeza não *Certainly not*	Provavelmente não *Probably not*	Talvez *Maybe*	Provavelmente *Probably*	Com certeza *Certainly*
17.Meu (minha) filho (a) aceitaria provar uma hortaliça com aparência diferente do que está acostumado (a) a ver *My child would accept to taste a vegetable that looks different from what he/she is used to see*	Com certeza não *Certainly not*	Provavelmente não *Probably not*	Talvez *Maybe*	Provavelmente *Probably*	Com certeza *Certainly*
18. Você acha que ele/ela provaria uma hortaliça que ele/ela nunca provou antes? *Do you think he/she would taste a vegetable that he/she never* *tried before?*	Com certeza não *Certainly not*	Provavelmente não *Probably not*	Talvez *Maybe*	Provavelmente *Probably*	Com certeza *Certainly*
19. Na casa de um amigo, você acha que ele/ela provaria uma hortaliça nova? *At a friends’ house, do you think he/she would taste a new vegetable?*	Com certeza não *Certainly not*	Provavelmente não *Probably not*	Talvez *Maybe*	Provavelmente *Probably*	Com certeza *Certainly*
20. Na escola, você acha que ele/ela provaria uma hortaliça nova? *At school, do you think he/she would taste a new vegetable?*	Com certeza não *Certainly not*	Provavelmente não *Probably not*	Talvez *Maybe*	Provavelmente *Probably*	Com certeza *Certainly*
21. Em casa, você acha que ele/ela provaria uma hortaliça nova? *At home, do you think he/she would taste a new vegetable?*	Com certeza não *Certainly not*	Provavelmente não *Probably not*	Talvez *Maybe*	Provavelmente *Probably*	Com certeza *Certainly*
22. Na escola, supondo que os amigos dele (a) aceitem a comida oferecida, meu (minha) filho (a) provaria a comida com a frequência a seguir: *At school, assuming his/her friends accept the food offered, my child would taste the food according to the following frequency:*	Nunca *Never*	Raramente *Rarely*	Às vezes *Sometimes*	Constantemente *Frequently*	Sempre *Always*
23. Em sua casa, considerando que os (as) amigos (as) de seu (sua) filho (a) aceitem as preparações oferecidas, seu (sua) filho (a) aceitaria essas preparações de acordo com a frequência a seguir: *At your home, considering that your child’s friends accept the preparations offered, your child would accept these preparations according to the following frequency:*	Nunca *Never*	Raramente *Rarely*	Às vezes *Sometimes*	Constantemente *Frequently*	Sempre *Always*
24. Na casa de um (a) amigo (a), considerando que os (as) amigos (as) dele (a) aceitem as preparações oferecidas, meu (minha) filho (a) provaria essas mesmas preparações de acordo com a frequência a seguir: *At a friend´s home, considering that his or her friends accept the preparations offered, my child would try these same preparations according to the following frequency:*	Nunca *Never*	Raramente *Rarely*	Às vezes *Sometimes*	Constantemente *Frequently*	Sempre *Always*
25. Em eventos (festas, reuniões), considerando que os (as) amigos (as) dele (a) aceitem as preparações oferecidas, meu (minha) filho (a) provaria essas mesmas preparações de acordo com a frequência a seguir: *At parties or meetings, considering that his/her friends accept the preparations offered, my child would try these preparations according to the following frequency:*	Nunca *Never*	Raramente *Rarely*	Às vezes *Sometimes*	Constantemente *Frequently*	Sempre *Always*
